# Effect of Diet and *Helicobacter pylori* Infection to the Risk of Early Gastric Cancer

**DOI:** 10.2188/jea.13.162

**Published:** 2007-11-30

**Authors:** Sang-Ah Lee, Daehee Kang, Ki Nam Shim, Jae Won Choe, Weon Seon Hong, Haymie Choi

**Affiliations:** 1 Department of Food and Nutrition, Seoul National University.; 2 Department of Preventive Medicine, Seoul National University College of Medicine.; 3 Department of Internal Medicine, University of Ulsan College of Medicine, Asan Medical Center.

**Keywords:** early gastric cancer, Helicobacter pylori, salt intake

## Abstract

BACKGROUND: The association of dietary habits and *Helicobacter pylori* infection with early gastric cancer is still unclear.

METHODS: A hospital-based case-control study was conducted in Korea. Sixty-nine patients were newly diagnosed as having early gastric cancer at the Division of Gastroenterology, Asan Medical Center, and 199 healthy subjects who visited the Health Promotion Center of the this same hospital for annual health examinations were selected as controls. *Helicobacter pylori* infection status was assayed by ELISA, and information for dietary habits was obtained by interview using a semi-quantitative food frequency questionnaires. Preference for salty taste was also evaluated using a sensitive test.

RESULTS: *H. pylori* seropositivity was observed in 88% of cases, as compared with 75% of controls (OR=5.3, 95% confidence interval:1.7-16.5). Adaptive salt concentration was significantly and positively associated with early gastric cancer risk (p<0.01). Decreased risks of early gastric cancer were observed in association with intakes of clear broth, raw vegetables, fruits, fruit or vegetable juices, and soybean curds. On the other hand, a high intake of salt-fermented fish and kimchi were associated with an elevated risk of early gastric cancer. Subjects with positive *H. pylori* infection and a high salty preference had a 10-fold higher risk of early gastric cancer than subjects without *H. pylori* infection and with a low salty preference (p for interaction = 0.047).

CONCLUSION: Some dietary factors and *H. pylori* infection are significantly associated with early gastric cancer. In particular, high-salty diets may enhance the effect of *H. pylori* infection in gastric carcinogenesis.

The incidence of gastric cancer has been gradually decreasing over the past 50 years in the world.^1^ However, it is the leading cause of cancer-related deaths for both sexes in Korea, and mortality rate of gastric cancer was 23.9 per 100,000 population in 1998,^2^ which was the second highest in the world.^1^ Although early gastric cancer differs from advanced gastric cancer regarding to the prognosis and tumor stage,^3^ epidemiologic evidence supports the hypothesis that most cases of untreated early gastric cancer will progress to advanced gastric cancer within 4 or 5 years.^2^^,^^4^ The reason why early gastric cancer was studied was that more valid dietary information was able to be obtained from early gastric cancer patients, and that *Helicobacter pylori* infection was detected more commonly in early gastric cancer patients than in advanced gastric cancer cases because of a loss of bacterial colonization in progressively atrophic stomach.^5^

The results of epidemiologic studies suggest that occupation, family history, life-style factors such as smoking, alcohol drinking, and dietary factors, and socio-economic status are associated with the risk of gastric cancer.^5^^-^^10^ Another major risk factor is infection with the gastric bacterium *H. pylori*, which colonizes the human stomach adjacent to gastric epithelial cells.^11^

As regards dietary factors, epidemiologic studies have reported an increased risk of gastric cancer with the ingestion of preserved, often salty foods or with a preference for salty foods.^12^ High-salt diets are known to contribute to gastric atrophy and to synergize with *H. pylori* infection through hyperplasia and further *H. pylori* colonization in animals.^13^ Relatively few studies have been conducted to evaluate the association with of salt intake, *H. pylori* infection, and dietary factors with early gastric cancer. Therefore, we undertook a case-control study to evaluate the preference for salty taste by using a sensitive evaluation test in subjects, and to assess the individual effects of dietary factors and *H. pylori* infection.

## METHODS

Seventy-two early gastric cancer cases were identified at the Division of Gastroenterology, Department of Internal Medicine, Asan Medical Center, Seoul, Korea, between March and September 1999. Patients newly diagnosed by endoscopy without previous gastric surgery were categorized as early gastric cancer cases. During the same period, 215 healthy subjects visited the Health Promotion Center of the same hospital for annual health examinations, were recruited as the potential controls. The control subjects had no abnormal gastric conditions examined by endoscopy or upper gastrointestinal series and no other known cancer or systemic disease. Subjects without *H. pylori* infection data nor dietary information were also excluded. A total of 69 cases and 199 controls were included in this study. Informed consent was obtained before the interview, but the Institutional Review Board approval was not obtained because the committee was not set up when the study was conducted.

Serum *H. pylori* antibody was measured by ELISA using HP™(Chong Kun Dang Pham. Corp., Korea) for all the subjects. Preference for salty taste was assessed using diluted rice gruels containing 0.1, 0.3, 0.5, 0.7, and 0.9% NaCl after semi-quantitative food-frequency questionnaire (FFQ) was done. The five different diluted rice gruels were arranged randomly and the subjects were asked to select a preferred concentration.

Two to three weeks after the diagnosis was made, person-to-person interview was carried out on dietary habits during the preceding year. The interview was worked out using semi-quantitative FFQ of which validity was evaluated in a previous study.^14^ In the result of semi-quantitative FFQ validation study, in which the questionnaire was compared with the 24 hour recall method, kappa values of energy, protein, fat, and carbohydrate intake were 0.82, 0.56, 0.47, and 0.69, respectively.^14^ Pictures of actual size for one serving size were used to help participants estimated their usual serving portion sizes. The semi-quantitative FFQ consisted of 24 food groups with 161 food items. The individual 161 foods items that suspected of being risk or protective factors in gastric cancer on the basis of previous studies^5^^,^^7^^-^^9^ were selected. The food groups were classified according to cooking methods of Korea, and the consistent of the food groups were as follows: rice, clear broth (prepared with vegetables, seafood, soybean curds, or beef with 50-60% water of total dish), soybean paste broth, soybean paste stew, other stew, salt-fermented fish, kimchi (preferred with salt-pickled and fermented vegetables seasoned with red pepper, onion, garlic, and so on), seasoned raw vegetables (seasoned with red pepper, garlic, onion, vinegar, and so on), raw vegetables, fruits, milk and milk products, tea, coffee, noodles, fish, pork, beef, chicken, potato, soybean curd, cooked seasoned vegetables, breads, nuts, and seeds. The seasonal fruit intakes were evaluated from the frequency during the fruit production season and converted to the frequency for 1 year (e.g., if the production period of orange is 6 month and the frequency of the orange is 5 or 6 times a week, the frequency of orange is 2 or 3 time a week for 1 year). General characteristics of the subjects (e.g. age, sex, education, and income), family history of gastric cancer, cigarette smoking, and alcohol drinking were also included in the semi-quantitative FFQ.

Unconditional logistic regression models were used to estimate odds ratios (ORs) and their 95% confidence intervals (CIs). Multiple logistic regression models were used to evaluate the relation between each of the dietary factors and early gastric cancer risk after adjusting for age, sex, family history, duration of education, and *H. pylori* infection. The consumption of each food group was categorized into three levels using tertile cut- off points except for soybean curd, salt-fermented fish, and kimchi. For the joint distribution of *H. pylori* serostatus and salt preference were assessed by adding multiplicative interaction product in the final model. All the analyses were carried out with the SPSS^®^ (version 10.0) statistical software package.

## RESULTS

**Figure 1.**
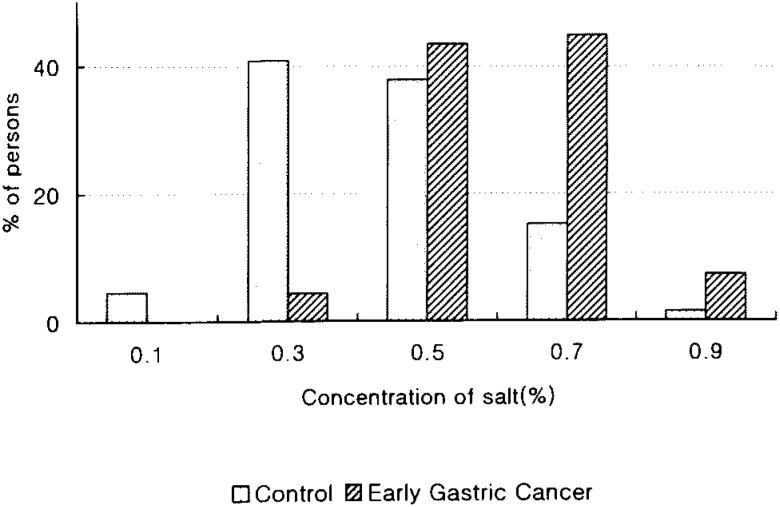
Distribution of preference for salty taste between early gastric cancer and controls

Table 1 shows the distribution of cases and controls according to age group, sex, education, family history of gastric cancer, smoking, alcohol drinking, and *H. pylori* infection. Family history of gastric cancer (OR=11.6, 95% CI: 4.3-31.8), and *H. pylori infection* (OR=5.3, 95% CI: 1.7-16.5) were significantly associated with an increased risk of early gastric cancer. The ORs were estimated by unconditional logistic regression models adjusting for age and sex. Moreover, the ORs of early gastric cancer increased dose-dependently as the number of cigarettes smoked per day (p for trend <0.01). Early gastric cancer cases were found to have preference for high salty taste compared to the control group (p<0.01, Figure 1).

**Table 1.** Odds ratios of early gastric cancer in relation to age, gender, socioeconomic status, family history, life-styles, and H. pylori infection.

	mic staearly gastric cancer (n=69)	control (n=199)	OR* (95%CI)
Age(yrs)			
≤40	6 ( 9)	35 (18)	
41-55	19 (28)	123(62)	
≥56	44 (64)	41 (21)	

Sex			
Female	19 (28)	83 (42)	
Male	50 (73)	116 (58)	

Education (yrs)			
≤10	31 (49)	33 (17)	1.0
10-13	16 (25)	52 (27)	0.1 (0.01-0.6)
≥14	16 (25)	107 (56)	0.5 (0.4-0.7)
missing**	6	7	

Family history			
No	48 (73)	178 (93)	1.0
Yes	18 (27)	13 ( 7)	11.6 (4.3-31.8)
missing**	3	8	

Smoking			
No	25 (36)	106 (53)	1.0
11-20/day	28 (41)	71 (36)	1.3 (0.5-3.3)
≥21/day	16 (23)	22 (11)	3.1 (1.0-9.5)

Drinking			
No	20 (29)	73 (37)	1.0
≤2-3/week	30 (61)	104 (83)	2.3 (0.7-8.0)
≥4-5/week	19 (39)	22 (18)	1.1 (0.5-2.8)

*H. pylori* infection			
No	8 (11)	50 (25)	1.0
Yes	61 (88)	149 (75)	5.3 (1.7-16.5)

* Adjusted for age, sex, education, family history of gastric cancer, smoking, alcohol drinking, and *H. pylori* infection.

** Missing data were substituted in the multivariate analysis.

OR: odds ratio.

CI: confidence interval.

Percentages in parentheses.

Eight of the 24 food groups studied were found to be significantly associated with early gastric cancer (Table 2). A significant dose-dependent decrease in the early gastric cancer risk was associated with the increased intake of clear broth, seasoned raw vegetables, raw vegetables, fruits, and fruit or vegetable juices (p for trend<0.01), and soybean curd (OR=0.3, 95% CI: 0.2-0.8). On the other hand, a significant increase in the early gastric cancer risk was observed with increased intake of salt-fermented fish (OR=2.4, 95% CI: 1.0-5.7) and kimchi (OR=1.9, 95% CI: 1.3-2.8).

**Table 2.** Odds ratio of early gastric cancer risk related with different type of food by multiple logistic analysis.

Food	early gastric cancer (n=69)	control (n=199)	adjusted OR* (95% CI) p value for trend
Clear broth			
<l/week	49 (71)	97 (49)	1.0
1-3/week	14 (20)	58 (29)	0.4 (0.2-0.9)
>3/week	6 ( 9)	44 (22)	0.2 (0.1-0.8)
			p=0.01
Seasoned raw vegetables			
2/month	27 (39)	49 (25)	1.0
2-7/month	25 (36)	67 (34)	0.4 (0.2-0.9)
>7/month	17 (25)	83 (42)	0.2 (0.1-0.6)
			p=0.01
Raw vegetables			
<4/week	52 (75)	74 (37)	1.0
4-6/week	7 (10)	52 (26)	0.2 (0.1-0.5)
>6/week	10 (15)	73 (37)	0.2 (0.1-0.5)
			p<0.01
Fruits			
<3/week	47 (68)	80 (40)	1.0
3-5/week	10 (22)	41 (21)	0.4 (0.2-1.1)
>5/week	12 (13)	78 (39)	0.3 (0.1-0.7)
			p<0.01
Fruit or vegetable juices			
<2/month	45 (65)	84 (42)	1.0
2-9/month	15 (22)	53 (27)	0.6 (0.3-1.1)
>9/month	9 (13)	62 (31)	0.5 (0.2-1.2)
			p<0.01
Soybean curd			
<1/month	51 (74)	94 (47)	1.0
≥l/month	18 (26)	105 (53)	0.3 (0.2-0.8)

Salt-fermented fish			
<1/month	22 (32)	177 (89)	1.0
≥l/month	47 (68)	22 (11)	2.4 (1.0-5.7)

Kimchi			
<2/day	19 (28)	112 (56)	1.0
≥2/day	50 (73)	87 (44)	1.9 (1.3-2.8)

Adjusted for age, sex, education, family history of gastric cancer, smoking, drinking, and *H. pylori* infection.

OR: odds ratio.

CI: confidence interval.

Percentages in parentheses.

Subjects with positive *H. pylori* infection and a high salty preference had a 10.1-fold higher risk of early gastric cancer than subjects without *H. pylori* infection and with a low salty preference (p for interaction = 0.047) (Table 3).

**Table 3.** Odds ratios* for the interaction between *H. pylori* infection and a preference for salty food on early gastric cancer

		*Helicobacter pylori* infection	
		Negative	Positive
	≤0.3% NaCl		
Preference for salty food		1.0(reference)	1.7 (0.6-4.7)
		[3/37] ^†^	[28/119]

	>0.3% NaCl	1.4(0.2-8.6)	10.1 (3.4-30.0)
		[3/10]	[33/23]

* Adjusted for age, sex, family history of gastric cancer, education, smoking, and drinking

^†^ [number of cases / number of controls], p for interaction =0.047.

95% confidence intervals in parentheses.

## DISCUSSION

This hospital-based case-control study found that *H. pylori* infection, some dietary factors, and a salt preference were significantly associated with the risk of early gastric cancer. Dietary salt has been linked to gastric cancer risk in numerous epidemiologic studies.^1^^,^^15^^-^^17^ Morbidity and mortality rates of gastric cancer remain high in Asia, where the salt intake is the highest in the world.^1^^,^^15^^-^^17^ The estimation and measurement of salt intake of individuals are difficult and subject to many potential errors. Previous studies as having used various approaches including (1) comparisons of the intakes of highly salted foods; (2) estimation of salt intake from appropriate food composition tables; (3) interview assessment preference for a salt taste; and (4) analysis of 24 hour urinary sodium excretion.^16^ In the present study, the difference in the salt consumption between cases and controls was not clear when salt intake was estimated from the semi-quantitative FFQ data (data not shown). However, it was demonstrated that the early gastric cancer group preferred to salty taste more than the controls, by the described preference test for salty taste. The assessment of salt intake on the basis of semi-quantitative FFQ data using the food composition table probably suffered much inaccuracy, and thereby necessarily underestimating the true risk of early gastric cancer associated with salt consumption.

A high intake of salt-fermented fish, i.e. salted fish and shellfish by fermenting for several weeks or months, was found to be associated with an increased risk of early gastric cancer. An increased risk of gastric cancer associated with the intake of smoked or salted foods has been documented by several studies. The present finding additionally suggests a role for nitrite and salt in the pathogenesis of gastric cancer.^16^ It has been suggested that salt intake is strongly associated with intestinal metaplasia and that this potentiates the effects of carcinogens.^18^ The incidence of gastric cancer is clearly higher in countries where diets are rich in salt.^19^

The present findings on vegetables and fruits are in agreement with the current knowledge. It has consistently been reported that high consumption of fruit and vegetables is associated with decreased risk of gastric cancer in many countries with diverse dietary habits.^20^

Non-fermented soy products, such as tofu, soymilk, and rice with soybean, were related to decrease in the risk of early gastric cancer. On the other hand, fermented soy paste showed no difference between the early gastric cancer and control groups (data not shown). In a previous case-control study in Korea, tofu showed a protective association with gastric cancer, but fermented soyfoods were found to be associated with an increased risk.^5^ Reviewing literature on soy and cancer, Messina et al.^21^ noted that there was an inconsistent relationship between the intake of soyfoods and gastric cancer, primarily because the risk seemed to be elevated in relation to the intake of fermented soyfoods, although a decreased risk with the intake of non-fermented soyfoods were shown. Soybeans are an abundant source of isoflavones, which are antioxidants, and possess other antitumor activities, including the inhibition of angiogenesis, topoisomerase, and tyrosine kinase.^22^

The frequency of kimchi intake was found to be positively related with early gastric cancer in our study. A previous study, upon 213 cases of gastric cancer and the equal number of controls, indicated that the consumption of pickled vegetables (kimchi and pickled radish root) was associated with an increased risk in Korea.^7^ On the other hand, Kim et al. recently reported that the risk of gastric cancer was low among those with a high consumption of kimchi in a case-control study with 136 cases and matched controls.^23^ Thus, the effect of kimchi on gastric cancer still has remains controversial in studies among Korea population.

Our finding of decreased early gastric cancer risk was also related to clear broth intake, which might be due to the low salt content in clear broth. However, whether this effect was due to low salt concentration or to other food components should be explored further.

*H. pylori* infection is generally considered to be associated closely with the development of early gastric cancer. In the present study, its prevalence in the control group was 75%, which is similar to that found in previous studies in the Korean population.^24^ Moreover, in the present study, the interactive effect between *H. pylori* infection and a preference for salty taste in early gastric cancer was statistically significant, which suggests that high-salty diets may synergize with gastric *H. pylori* infection, and increase the risk of early gastric cancer. The accumulated findings of several epidemiologic studies indicated that both salty foods and *H. pylori* infection are closely linked to gastric carcinogenesis.^21^ In addition, the consumption of pickled vegetables was positively associated with the prevalence of *H. pylori* infection in a Japanese study.^17^ This study also suggested that the relationship between *H. pylori* infection and gastric cancer may be confounded by salt consumption. Importantly, in relation to *H. pylori* infection, dietary factors appear to be vital component for future studies.

In the present study, a family history of gastric cancer was positively associated with early gastric cancer, which is consistent with the findings of other studies,^7^^,^^8^ but does not necessarily indicate a genetic predisposition. Cigarette smoking was also associated with the increased risk of early gastric cancer with a dose-dependent manner. The finding is entirely consistent with the results of a meta-analysis of tobacco and gastric cancer, which gave an overall estimated of relative risk of 1.5 to smokers versus non-smokers.^25^

The present study has several strengths. Early gastric cancer patients selected in the study were diagnosed by gastroscopy, which was probably reduced the changes of dietary habit influenced by recognition of disease conditions. In addition, *H. pylori* infection was detected more commonly in patients with early gastric cancer than in those with advanced cases, possibly because of a loss of the bacterial colonization in progressively atrophic stomach.^5^ The study incorporated an accurate and unbiased assessment of taste preference for salt using a sensitive evaluation method. The study evaluated an interaction between salty taste preference and *H. pylori* infection.

Although the current study contains several limitations (e.g., small sample size, a hospital-based study, and so on), we did not anticipate these potential biases might affect the results. First, potential confounders (e.g., age, sex, education, and so on) were adjusted by the multiple logistic analyses. Second, non-differential misclassification of *H. pylori* infection between cases and controls might underestimate the results. However, a hospital-based approach may not provide the generalizability of the findings observed in this study.

In conclusion, the results of the study suggest that some dietary factors and *H. pylori* infection are significantly associated with early gastric cancer, and that high-salty diets may synergize with gastric *H. pylori* infection and increase the risk of early gastric cancer.

## Appendix

List of food and food groups used in semi-quantitative food frequency questionnaire

**Table:**

Foods	Item
Rices	Rice, Rice with grains (2)*
Clear broth	Bean sprout, Sea mustard, Beef shank, Fish paste (4)
Soybean paste broth	Chinese cabbage, Spinach, chard, stalk of sweet potato, Potato, Dubu, Potato+Dubu, Potato+Dubu+squash (8)
stew	Ham+Sausage+Bacon, Uncurdled soybean curd, Damduk, Roe egg, Kimchi (5)
Seasoned fermented	Galic (vineger, soybean broth), Radish (3)
Soybean paste stew	Dubu, Potato, Dubu+potato, Dubu+potato+meat (4)
Salt-fermented fish	Beka squid, Roe egg, Shad, Small shrimp, Gill, Squid, Clam (6)
Kimchi	Chinese cabbage, Radish leaves, Radish roots and leaves, Cubed radish, Cucumber (5)
Seasoned raw vegetable	Radish, Chinese cabbage, Lettuce, Cucumber, Root of Chinese bellflower (5)
Raw vegetable	Head lettuce, Lettuce, Cabbage, Cucumber, Chinese cabbage, Pepper, Carrot, Onion, Perilla leaves (8)
Fruit	Persimmon, Citrus, Strawberry, Melon, Banana, Peach, Apple, Watermelon, Orange, Grapefruits, Muskmelon, Cherry tomato, Kiwi, Tomato, Pineapple, Grape (16)
Milks	Milk, Yoghurt, Soybean milk (3)
Tea	Green tea (1)
Coffee	Roasted beans, Regular, add sugar, add cream, add sugar and cream (5)
Noddle	Noddle in broth, Seasoned noddle, Black bean paste noddle, Ramyon, Ddokguk, Mando and broth, Hand made noddle (7)
Fish	Flat fish, Hair fish, Mackerel, Pacific saury, Yellow croaker (5)
Pork	Rib, Shoulder, Belly, Stew of rib (4)
Beef	Grilled of beef, Rib, Loin, Stew of rib, Pan-fried beef roll (5)
Chicken	Egg, Fried chicken with spicy sauce, Chicken broth with red pepper sauce, Fried chicken, Plain chicken broth (5)
Potato	Stir-fried, Pan-fried, Braids, Fried
Soybean curd	Pan-fried, Braids
Cooked seasoned vegetable	Mungbean sprout, Soybean sprout, spinach, Mixed dish of vegetable and beef, squash (5)
Bread	Doughnuts, Roll cake, Muffins, Dock marked bread, Small red bean jam bread, Loaf bread
Nuts and seeds	Peanuts, Almonds, Gingko nuts, Pine nuts

* The number of food items in parentheses.
